# Diseases of the Nails

**Published:** 1907-08-24

**Authors:** 


### Dermatology.

### DISEASES OF THE NAILS.

It may be that affections of the nails are unattractive, or that they give rise to little or no suffering, or that their treatment often proves unsatisfactory; nevertheless, the fact remains that these useful appendages and their diseases have received but scant attention alike from clinical observers and the writers of dermatological treatises. Nails are only modified epidermis, after all, and therefore we might expect them to share more frequently in the disorders of that tissue than they actually do; but owing to their resistant structure they appear to enjoy a sort of immunity from many skin affections proper.

Putting aside such trifles as white spots, made much of by the superstitious, the presence or absence of the lunula, and the transverse ridges or grooves marking the occurrence of some illness, the most important morbid conditions of the nails met with in practice are those affecting their polish, shape, and texture. Strong, highly polished nails, devoid of grooves or spots of any kind, are generally associated with bodily vigour, but they have also been observed in the gouty (Hutchinson).

### Abnormal Growth.

A given finger-nail is said to be completely renewed in 124 days upon an average, the exact time depending upon the time of year and the general health. In very neglected cases genuine hypertrophy may occur, so that the nail degenerates into a veritable claw, forming, when the horny growth is much exaggerated, the condition known as onychogryphosis, which is not uncommonly seen affecting the great toe-nails of elderly persons. The opposite condition is one of extreme brittleness, the nail-plate being at the same time reduced to a thin film. Small pieces of the nail are continually being chipped off as the result of slight injuries, catching in the clothes, etc., and tearing may occur, leading to bruising and soreness of the matrix. At other times the whole substance of the nail seems little more than a mass of densely packed fibres, which easily become separated from one another, causing an unpleasant roughness of the nail. When a disease is limited entirely to the nails the diagnosis is "little more than guesswork." Fortunately there are nearly always some indications upon the skin which are a guide, at any rate, to the true condition affecting the nails.

Atrophy of the nails may occur after an acute illness, especially one of the exanthemata, and their direction of growth and texture may both be altered considerably. Several cases of "spoon-nail" (koilonychia) have been shown of recent years before the dermatological societies in which the nail-plate is concave upwards, presenting a very curious appearance. Most of the above nail troubles are associated with some constitutional weakness, and arsenic given internally is often of great value.

### Ringworm of the Nails.

The ringworm fungus sometimes attacks the nails (onychomycosis) either alone or together with some other part of the cutaneous system. The disease is frequently missed, as the subjective symptoms do not always lead the sufferer to seek advice. The nails lose their lustre, and become thickened, dull and brittle. Part of the nail-plate may scale off, revealing a roughened, disintegrated surface. Without a microscopic examination of scrapings taken from the nail and mounted in potash solution the diagnosis can scarcely be made at all. A case of tinea unguium was recently exhibited by Dr. Graham Little before the Dermatological Society, in which all the nails of both hands and feet had been thus affected for fourteen years! Considering how frequently children with ringworm of the scalp must scratch, and therefore, presumably, infect the nails with the fungus, it is surprising that disease of the nails is not more often observed. Adults are, perhaps, affected more than children.

Removal of the nail under an anæsthetic is a radical cure, but if this is impossible the best plan is to scrape the nail thin with a file or a piece of glass, after which a parasiticide ointment, such as chrysarobin or ammoniated mercury, may be applied. What is known as "Harrison's treatment" is perhaps the best and the most universally applicable. It consists in covering the affected nails with pieces of lint soaked in a solution of potassium iodide in potash for fifteen minutes, followed by a similar application of lint soaked in a solution of perchloride of mercury in spirit for twenty-four hours. Indiarubber finger-stalls may be worn throughout the treatment, which is cleanly and easy to carry out. The formula of the two solutions is thus given:—

No. I.—Potash Solution:—

| | |
|---|---|
| Potass. Iodidi ... ... ... | ʒj |
| Liquor. Potassæ ... ... ... | |
| Aq. Dest. aa ... ... ... | ℥ss |

No. II.—Mercury Solution:—

| | |
|---|---|
| Hydrargyri Perchloridi ... ... | iv grs. |
| Spirit. Vini Rectif. ... ... ... | |
| Aq. Dest. aa ... ... ... | ℥ss |

### Psoriasis and Eczema of the Nails.

It is convenient to consider these two affections together not only because they are comparatively common, but on account of their similarity and difficulty of diagnosis. In both affections the nail-plate may be lifted somewhat away from its bed, and may undergo a certain amount of thickening. The adjacent skin about the finger-tips may be affected with the disease also, and if this be the case the diagnosis is easier. Small, black depressions are apt to occur in psoriasis of the nails, and irregular grooving is more usually seen in chronic eczema. Discoloration of the nails is met with in psoriasis, but it is also a feature of ringworm. The best applications are ointments of tar and salicylic acid, which should be kept continuously applied to the nails enclosed in appropriate finger-stalls. Such conditions as onychia (purulent inflammation of the matrix) and ingrowing toe-nail must be treated wholly on general surgical principles.

